# Study on the Livelihood Vulnerability and Compensation Standard of Employees in Relocation Enterprises: A Case of Chemical Enterprises in the Yangtze River Basin

**DOI:** 10.3390/ijerph17010363

**Published:** 2020-01-05

**Authors:** Xu Zhao, Chen Chi, Xin Gao, Yuefang Duan, Weijun He

**Affiliations:** 1 School of Economics and Management, China Three Gorges University, Yichang 443000, China; zhaoxu@ctgu.edu.cn (X.Z.); heweijun1519@ctgu.edu.cn (W.H.); 2 Research Center for Reservoir Resettlement, China Three Gorges University, Yichang 443000, China; peter_yf@aliyun.com; 3 Business School, Hohai University, Nanjing 211100, China

**Keywords:** enterprise relocation, livelihood vulnerability, sustainable livelihood framework, compensation standard, Yangtze River Basin

## Abstract

The relocation of chemical enterprises along the Yangtze River a necessary means of ecological protection in the Yangtze River Basin. Vulnerability assessment provides a new idea for the study of livelihood ability and compensation standard of employees after relocation. Based on the framework of “Exposure-Sensitivity-Adaptability” proposed by the Intergovernmental Panel on Climate Change (IPCC) and the survey data of 410 employees of relocation enterprises in the Hubei Province of the Yangtze River Basin, this study firstly constructs a livelihood vulnerability evaluation index system and evaluation model, and analyzes whether the employees of relocation enterprises have the ability to cope with the risk impact brought by the Yangtze River Ecological Restoration policy. Then, we use multiple linear regression model to explore the relationship between the group’s exposure, sensitivity, adaptability and livelihood vulnerability. Finally, we design a new compensation standard calculation method for special groups from the perspective of social cost, to alleviate their livelihood vulnerability and provide a theoretical basis and decision support for the government and enterprises to formulate and implement relevant resettlement standards. The results show that: (1) employees of all ages show a certain degree of vulnerability in their livelihood; (2) there are differences in livelihood vulnerability between male and female employees; (3) compared with other positions, the livelihood vulnerability of producers is relatively high, and the vulnerability index is unevenly distributed and internally differentiated; (4) a low family burden ratio, high education, convenient living conditions and complex social network can effectively reduce the vulnerability of employees’ livelihood; (5) the key obstacle factors affecting the sustainable livelihood of families are living convenience, adaptability to relocation, policy understanding, children’s burden ratio, education, and annual income per capita; (6) the alternative opportunity cost method can be used as the basis to determine the compensation standard of the relocated employees, which can better reflect the compensation effect of the opportunity cost in the existing definition of international compensation mechanisms and realize the leap from concept to action.

## 1. Introduction

In recent years, with the rapid development of the global economy, the world’s natural environment is deteriorating. According to statistics, 50% of the wetlands on the earth are destroyed, 50% of the rivers are polluted, and the fresh water supply is 30% lower than 25 years ago [1]. Environmental problems are a growing concern in the international community [2]; many measures for environmental protection have been adopted around the world, such as the “European sewer” in Germany [3], and the environmental plan in the United Nations and World Bank [4].

China’s rapid economic development mainly depends on resource utilization and heavy industry development, which leads to a large number of pollution problems [5], especially in the Yangtze River Basin. With the rapid development of the chemical industry along the Yangtze River, a lot of sewage is discharged into the Yangtze River. Thus, the surge of pollutants in sewage seriously threatens the ecological safety of the Yangtze River Basin. Along the River, nearly 600 km of coastal pollution zone has been formed, about 60% of the water bodies are polluted, and a variety of heavy metals seriously exceed the standard limits. This leads to the dilemma of “chemical industry encircling around the river”.

In order to protect the water environment of the Yangtze River and promote the construction of ecological civilization, some special pollution control measures were taken for hundreds of chemical enterprises within 15 km of the Yangtze River to solve the problem of “chemical industry encircling the river”, and the policy of “close, reform, move and transfer” was implemented within a time limit. Not only that, in the Erhai Ecological Reserve of Yunnan Province, in the upper reaches of the Yangtze River, the “pollution interception and control project surround lake” was implemented in a water ecological protection area, and more than 2000 hotels and inns within 100 m of the boundary stake were forced to be closed down. In addition to the Yangtze River Basin, there are similar situations in other regions, for example, 15 urban steel plants, such as Shigang and Xuangang Iron and Steel Corporation, were shut down and transferred in the Hebei province; more than 1200 coal and other industrial and mining enterprises have been shut down and withdrawn from the Helan Mountain National Nature Reserve of Ningxia.

In these enterprising migrations for the purpose of “protecting the ecological environment and realizing sustainable development”, a large number of employees have to leave the original work place, resulting in difficulties such as diversion and laid-off. Their living conditions and livelihood patterns are inevitably impacted. For example, 134 chemical enterprises in Yichang along the Yangtze River involved 52,500 employees [6]; nearly 4000 workers of coal sandstone enterprises were influenced in the Helan Mountain Reserve of Ningxia [7]; thousands of residential houses were closed, and the income sources of all local tourism practitioners were cut off in the Erhai Lake [8]. We can find that the large-scale relocation of enterprises caused by ecological environment protection makes the relocated employees face the crisis of livelihood capital loss, reemployment difficulties and poverty caused by relocation. Sustainable livelihoods are facing the risk of breaking down, and they are likely to fall into a new livelihood dilemma [9].

Although local governments in China have successively introduced support measures for these employees, there is a widespread problem of “focusing on industrial transformation and upgrading, neglecting employees’ compensation and resettlement”, due to insufficient overall planning and top-level design in policy formulation. Local governments fail to consider the hidden losses and compensation, such as relocation and reassignment [10]. There are still many controversies in theory about the livelihood analysis of employees affected by the relocation of such enterprises, resulting in difficulties in the implementation of practice and lack of implementation of rules and specific schemes. Enterprises are relocated to protect of nature and the residential environment, so the employees involved encounter external forces such as forced relocation in the short term, which induces the impact vulnerability of livelihood capacity discomfort. Different from the groups affected by environmental protection in the past, this group were originally urban enterprise employees, but they were forced to move because of ecological protection; their interventional poverty risk is coupled with multiple social changes. Environmental protection policy, enterprise transformation and regional development are intertwined, which together affect the livelihood capital and livelihood ability of employees, which leads to the emergence of livelihood vulnerability of the relocated employees in the end.

The livelihood vulnerability refers to the unstable state of the individual or family in the process of livelihood development due to the change in livelihood structure or the impact of external forces [11]. Related to the study of livelihood vulnerability, Assan [9], Kumar [12] and Moser [13] have studied the formation mechanism of livelihood vulnerability of special groups and the key factors affecting their livelihood development; other foreign scholars have studied the construction of a vulnerability evaluation index system and model [14,15]. In contrast, scholars in China have mainly studied the assessment of livelihood vulnerability, which is divided into two parts: one is a measurement of the decline in family welfare level under the drastic change in environment with the interventionist poverty state [16], the other is to construct a unified assessment system containing the ecological environment change indicators, the economic indicators and the social indicators based on the “ Exposure-Sensitivity-Adaptability” framework proposed by the Intergovernmental Panel on Climate Change (IPCC) [17,18].

Although there has been some research on livelihood vulnerability, it still has the following limitations: (1) most scholars pay attention to the impact of environmental degradation on the livelihood of the agricultural population, but ignore the enterprise employees whose livelihood is damaged due to relocation for environmental protection; (2) as a special group, the former methods of livelihood vulnerability assessment are not suitable for the relocation of employees; (3) a lack of research on compensation standards for the livelihood vulnerability of relocated employees. Therefore, in order to fix these research gaps, this paper first measures the livelihood vulnerability of relocated employees, finds out the key factors that affect their livelihood vulnerability and sustainable livelihood ability, and tries to solve the livelihood vulnerability of relocated employees by designing a targeted compensation standard. This not only improves the international study of livelihood vulnerability, but also proposes a new compensation standard for special groups affected by ecological protection.

## 2. Research Framework

### 2.1. Research Area

The Yangtze River has a unique ecosystem, which is an important ecological treasure house in China. The ecological space, such as the shoreline of the river lake region, is an important part of the Yangtze River ecosystem. Hubei Province, located in the center of the Yangtze River Basin, has 1061 km of Yangtze River coastline, and is the province with the longest Yangtze River trunk line. Heavy chemical enterprises along the river are densely distributed with low technology and high energy consumption, which have caused serious air and water pollution for a long time. There are 21 chemical industry parks in Hubei Province, ranking second along the Yangtze River. According to statistics, in recent years more than half of the large-scale environmental events occurred in 11 provinces and cities along the Yangtze River [19]. Two major environmental pollution events occurred in Hubei Province in 2014. In June 2018, the Hubei provincial government vigorously promoted the special pollution control of chemical enterprises along the river. All existing chemical enterprises that do not meet the environmental protection requirements and have environmental pollution risks will be shut down or moved into the compliance areas, transformed and upgraded. Thus, hundreds of chemical enterprises are subject to “close, reform, move and transfer” within a time limit within the range of 1–15 km along the Yangtze River, involving nearly two hundred thousand employees who moved with them.

### 2.2. Research Framework

The large-scale relocation of high pollution enterprises caused by ecological environment protection has made a large number of urban employees lose job opportunities and encounter a series of livelihood crises. Their vulnerability can be explained by two reasons: first, their livelihood capacity is damaged. This means that people can’t continue to maintain their original livelihood when coping with the changes in the external livelihood environment, resulting in a weak and incompetent state. The second is the potential loss of livelihood capital, making livelihood vulnerability mainly the systematic livelihood risk caused by the lack of livelihood capital. However, the diverse livelihood choices and heterogeneous livelihood strategies in this group make it difficult for traditional theories and methods to effectively measure the degree of livelihood vulnerability and accurately assess the risk of intervention poverty [20].

**Figure 1:**
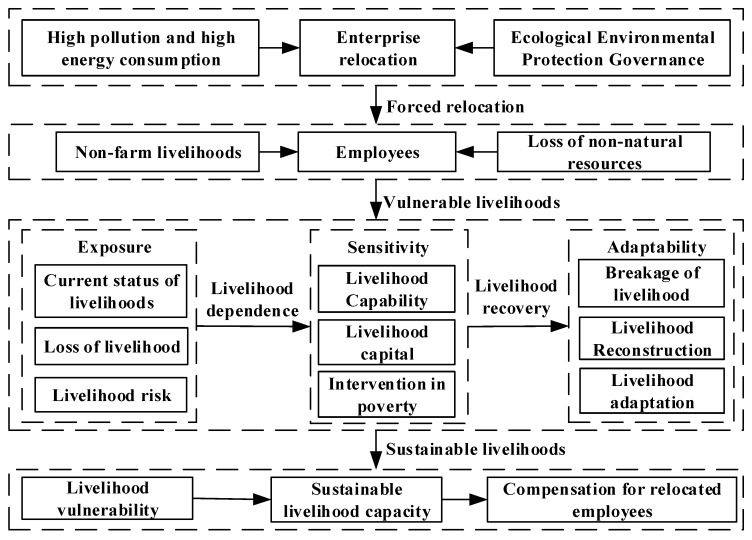
Research framework.

Therefore, at the micro level of the urban enterprise employees’ livelihood vulnerability, we can use the framework of “Exposure-Sensitivity-Adaptability” proposed by IPCC to assess whether the relocated employees have the ability to cope with the risk impact. At the same time, the response of the employees to policy evolution is fully considered. From the three aspects of employees’ exposure, sensitivity and adaptability, the evaluation index system and model of relocated employees’ livelihood vulnerability, under the background of environmental protection relocation, are constructed and used to quantify the impact of relocation policies on employees’ livelihood vulnerability. Then, the degree of obstruction model is used to measure the sustainable livelihood ability of different types of employees after relocation. Finally, a new compensation standard calculation method for this special group is constructed to alleviate their livelihood vulnerability, and to provide scientific and effective decision support for the design of relocation policy. The research framework is shown in Figure 1.

## 3. Method and Data Source

### 3.1. Livelihood Vulnerability Assessment

#### 3.1.1. Framework for Livelihood Vulnerability Analysis

The concept of “vulnerability” originated from the study of geological hazards in natural systems. In recent years, vulnerability research has extended from its initial focus on the vulnerability of natural environmental systems [21] to the vulnerability of human systems, especially the livelihood of specific populations in social–ecological coupling systems [22,23,24]. With the development of livelihood vulnerability, livelihood vulnerability has become an important analysis tool to analyze the instability and vulnerability of individual livelihood structure [17]. It can be analyzed from health and nutrition, poverty support, natural ecology, technical ability and other dimensions [25].

The theoretical framework of “Exposure-Sensitivity-Adaptability” proposed by IPCC has been widely used in livelihood vulnerability research at different scales, such as global, national, regional and social scales. They believe that livelihood vulnerability is calculated as follows [26]:

*Vulnerability* = *Risk* − *Adaptation* = *(Exposure + Sensitivity)* − *Adaptability* (1)

According to Equation (1), vulnerability includes three elements, which are exposure, sensitivity and adaptability. Exposure includes the extent to which groups are affected by environmental changes and other relevant impacts caused by environmental changes [27]. Sensitivity is a multidimensional quantitative response relationship between external stress and its consequences, as well as the degree of the positive or negative effects of stress on exposed groups [25]. Adaptability refers to the ability of a group to adjust itself to actual or expected environmental changes and respond to the results of environmental changes [28]. When exposure and sensitivity increase, vulnerability inside the system will increase, while adaptability can reduce vulnerability to a certain extent [29].

#### 3.1.2. Evaluation Index System of Livelihood Vulnerability

According to the framework of “Exposure-Sensitivity-Adaptability”, in the process of selecting indicators, this paper fully refers to the indicators of livelihood vulnerability in the previous classic literature and adjusts them in combination with the actual situation of the study area. On this basis, we construct the evaluation index system of livelihood vulnerability, which is listed in Table 1.

#### 3.1.3. Livelihood Vulnerability Assessment Model

At present, the most widely used model for assessing livelihood vulnerability is the comprehensive index assessment model [30]. This model can reflect the relative vulnerability of the assessed individuals accurately and effectively. It has become a more practical vulnerability research method in the world. This paper uses this model to measure the livelihood vulnerability of employees in relocation enterprises. The calculation formula is as follows

*LVI* = *(E + S)* − *A* (2)

where *LVI* is the Livelihood Vulnerability Index; *E + S* is the Risk Index; *E* is the exposure; *S* is the sensibility; *A* is the adaptability.

Firstly, the range standardization method is used to standardize the original quantified values of each index in order to eliminate the influence of different dimensions, magnitudes and variation ranges of survey data.

(3) $$\mathrm{Positive}\;\mathrm{indicators}:\;P_{ij}\;=\;\frac{x_{ij}-x_{i,min}}{x_{i,max}-x_{i,min}}$$

(4) $$\mathrm{Negative}\;\mathrm{indicators}:\;P_{ij}=\frac{x_{i,max}-x_{ij}}{x_{i,max}-x_{i,min}}$$

where *i* refers to the different indices, and *j* refers to the different employees. *P_ij_* represents the index *i*’s standardized value of employee *j* (*i* = 1, …, m; *j* = 1, …, n); *x_ij_* represents index *i*’s original value of employee *j*; *x_i,max_* and *x_i,min_* refers to the maximum and minimum values of each index *i*, respectively. Through standardization, all index values are between 0 and 1.

On this basis, the weight of each index is determined. The current methods of determining weights are mainly divided into subjective and objective methods. In order to avoid the influence of subjective and artificial factors, the principal component analysis method of objective weighting method is used to determine the weight of each index, to make the evaluation results more real [31]. The weights (*W_i_*) of each index under different dimensions are calculated, as shown in Table 1. The exposure (*E_j_*), sensitivity (*S_j_*) and adaptability (*A_j_*) of employee *j*’s livelihood were calculated by the weighted average method

(5) $$E_{j}=\frac{\sum_{i\;=\;1}^{a}\left(W_{Ei}\times P_{Eij}\right)}{\sum_{i\;=\;1}^{a}W_{Ei}},\;S_{j}=\frac{\sum_{i\;=\;1}^{b}\left(W_{Si}\times P_{Sij}\right)}{\sum_{i\;=\;1}^{b}W_{Si}},\;A_{j}=\frac{\sum_{i\;=\;1}^{c}\left(W_{Ai}\times P_{Aij}\right)}{\sum_{i\;=\;1}^{c}W_{Ai}}\;$$

where *P_Eij_*, *P_Sij_*, *P_Aij_* represents the index *i*’s exposure, sensitivity and adaptability of employee *j*’s livelihood; *W_Ei_*, *W_Si_*, *W_Ai_* represents the weight of indices *i* of exposure, sensitivity and adaptability; *a*, *b*, *c* represents the number of indices of exposure, sensitivity and adaptability, respectively. Combining Equations (1) to (5), we can get the livelihood vulnerability index of employees as follows

*LVI_j_* = *(E_j_ + S_j_)* − *A_j_* (6)

### 3.2. Sustainable Livelihood Ability Assessment Model

In order to improve the sustainable livelihood ability of relocated employees and reduce their livelihood vulnerability, it is necessary to find the factors that restrict the sustainable livelihood ability of relocated employees. Thus, we introduce the degree of obstruction model to measure the degree to which specific factors hinder sustainable livelihood capability [32]; the model for this is shown in Equations (7) and (8).

(7) $$\;O_{i}=\frac{I_{i}W_{Ai}}{\sum_{i\;=\;1}^{m}I_{i}W_{Ai}}\times 100\%\;$$

(8) $$\;I_{i}\;=\;1-P_{i}\;$$

where *O_i_* is the degree of hindrance of *i* to sustainable livelihood capacity. *I_i_* is the deviation of the index, the difference between *i* and the optimal value. *P_i_* is the standardized average value of *i*.

### 3.3. Compensation Model for Relocated Employees

In the process of enterprise relocation, the employees of relocated enterprises are forced to give up development opportunities (*OC*). In order to make up for the livelihood loss effectively and improve the sustainable livelihood ability of employees after relocation, these should be compensated. The compensation level should at least reach the minimum loss of employees due to enterprise relocation [33]. At the same time, the impact of enterprise relocation on employees’ own development is multifaceted; it not only affects the annual income of employees’ families, but also affects their work, social network and living convenience to varying degrees. Based on this, a comprehensive opportunity cost measurement model for the compensation of such special groups can be constructed to evaluate the value of social capital and study the relationship between compensation standard (CS) and development opportunity cost, which is shown in Equation (9).

*CS* ≥ *OC* = *OC*_1_ + *OC*_2_ (9)

where *OC*_1_ is the fixed social capital [34], which represents the social capital that does not change due to the relocation of the enterprise, such as education, and social network of relatives and friends; *OC*_2_ is the variable opportunity cost [35], which represents the opportunity cost that changes due to the relocation of the enterprise, such as annual income per capita, family consumption, residential convenience and household savings.

Social capital has the function of acquiring or exchanging labor force, information resources and emotional support [36]. By looking for the market cost of obtaining these resources, the value of social capital owned by employees can be approximately replaced [37]. Therefore, the calculation of *OC*_1_ can be expressed as follows

(10) $$\;OC_{1}=\overset{n}{\underset{i\;=\;1}{\sum }}OC_{1i}=\overset{n}{\underset{i\;=\;1}{\sum }}r_{i}\left(\theta *t_{i}+c_{i}\right),\;i=1,2,\ldots ,n\;$$

where *OC*_1*i*_ represents each component’s resource value of such social capital, *r_i_* represents the probability of employees using the resource value, *t_i_* represents the time paid, *θ* represents the wage rate (CNY/day), *c_i_* represents other costs. In technical resources (technical training, etc.) and the balance of human income and expenditure, the time value paid is not calculated, *t* = 0.

(11) $$\;OC_{2}=\overset{m}{\underset{j\;=\;1}{\sum }}\left(OC_{2j}-OC_{2j}^{\prime }\right),j=1,2,\ldots ,m\;$$

where *OC*_2*j*_ and *OC’*_2*j*_ represent the cost indicators of employees before and after relocation, respectively.

*OC*_2*j*_ is affected by different factors (*X_k_*). Based on the data before the relocation of the enterprise, the coefficient (*A_jk_*) of the influencing factor (*X_k_*) on each cost index level (*OC*_2*j*_) can be measured through regression analysis, which can be used to measure the *OC’*_2*j*_ of employees after the enterprise relocation

(12) $$\;OC_{2j}=f\left(X\right)\;$$

(13) $$\;OC_{2j}^{\prime }=\overset{p}{\underset{k\;=\;1}{\sum }}OC_{2j}A_{jk}\left(X_{k}-X_{k}^{\prime }\right),k\;=\;1,2,\ldots ,p\;$$

where k is the serial number of influencing factors, and $X_{k}^{\prime }$ is the value of influencing factors after relocation.

### 3.4. Data Source

**Figure 2:**
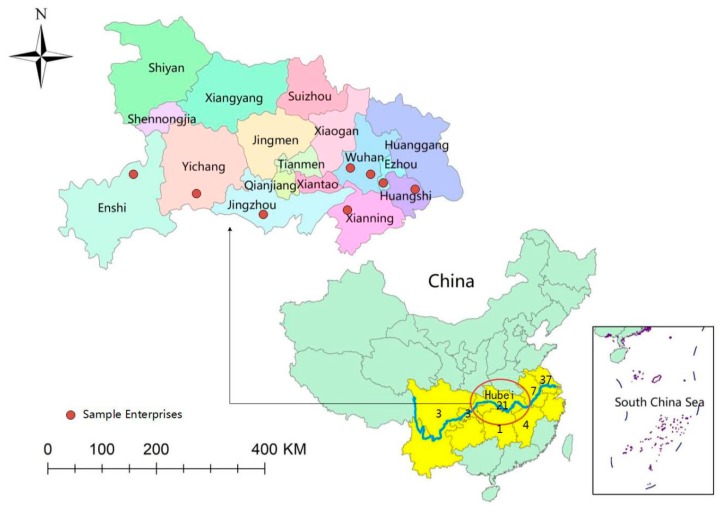
Locations of sample cities and enterprises.

The data for the questionnaire were collected from a random sampling survey conducted by the project leader among employees of chemical relocation enterprises in the Hubei Province of the Yangtze River Basin in November 2018. The survey site covers numbers of chemical enterprises in the range of 1–15 km along the river (Figure 2), such as Xingfa in Yichang and Jingtiandi in Jingzhou. The specific process of sampling is as follows: (1) collect a roster of all chemical enterprises in the research area, and number all enterprises in a certain order to form a complete list of total enterprises without repetition or omission, and take it as the first sampling frame of this survey; (2) determine the list of sampling enterprises by systematic sampling method in probability sampling; (3) collect the list of employees in the sample enterprises, and number them in turn as the second sampling frame; (4) the project investigator carries out simple random sampling in the numbered card that he took with, and the employees corresponding to each number are chosen to be interviewed face to face and fill in the questionnaire. Before investigation, the basic information and the implementation progress of “close, change, move and transfer” of the relocation enterprises were known from relevant government administrators and enterprise leaders. Finally, 430 questionnaires were sent out, and 410 valid questionnaires (95.3%) were obtained after removing outliers and invalid samples.

**Figure A1:**
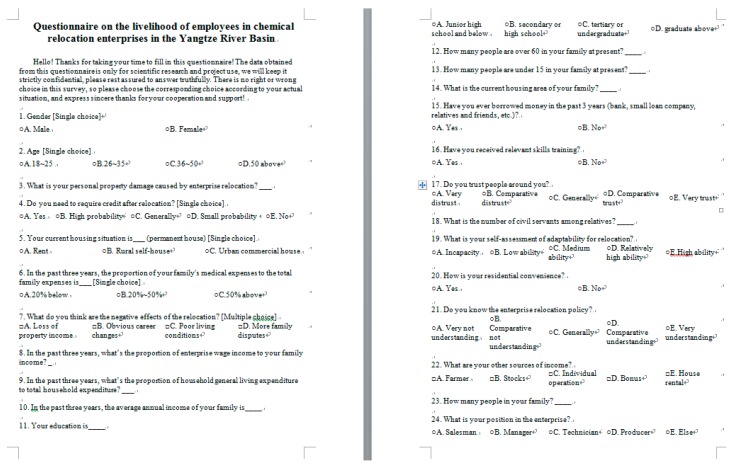
The questionnaire used in the study.

The survey involves: (1) the basic information of the relocated employees, including gender, age and position; (2) the exposure level of the relocated employees, including the situation of policy impact (property and other losses), and the possibility of credit and housing; (3) the sensitivity of the relocated employees, including health status, the negative impact of relocation policy on their lives; (4) the adaptability of the relocated employees, including material assets, credit assets, social networks, living convenience, self-assessment of adaptability and policy understanding. The questionnaire is shown in Appendix A (Figure A1).

## 4. Results

### 4.1. Descriptive Statistical Analysis

In general, the socio-economic characteristics of the sample are basically consistent with the employees’ situation. We asked supervisors in relocated enterprises, and the sample is representative. The descriptive statistical results of the questionnaires are shown in Table 2 and Table 3. According to the survey, 84.39% of employees are male and 15.61% are female, and more than 90% of employees are 26 to 50 years old. The distribution of gender and age is consistent with the actual situation of chemical enterprise. Among the sample, there are 40 salesmen, 54 managers, 48 technicians, 201 producers and 67 others. Producers and technicians are the backbone of enterprise development, accounting for a relatively high proportion of it, which is consistent with the actual personnel ratio of the enterprise.

There are some differences in family size, family burden ratio and per capita annual income of employees in different positions. At the grass-roots level of enterprise, producers’ per capita annual income is the lowest among the five groups, while their family number is large and the dependency burden is large.

At present, employment opportunities are scarce [38]. In the process of enterprise relocation, the means of livelihood for grass-roots employees is singular and their ability to deal with risks is weak [39]. Upon encountering unemployment or job transfer, livelihood activities are loose and the probability of livelihood risk is greatly increased. At the same time, to some extent, the relocation of enterprises will cause internal members of employees’ families to have big or small grievances. Therefore, the large scale of family means grass-roots employees need to face not only the external risk of policy impact, but also the internal risks to their family such as the conflict of kinship caused by the enterprise’s relocation.

### 4.2. Analysis of Employees’ Livelihood Vulnerability

**Figure A2:**
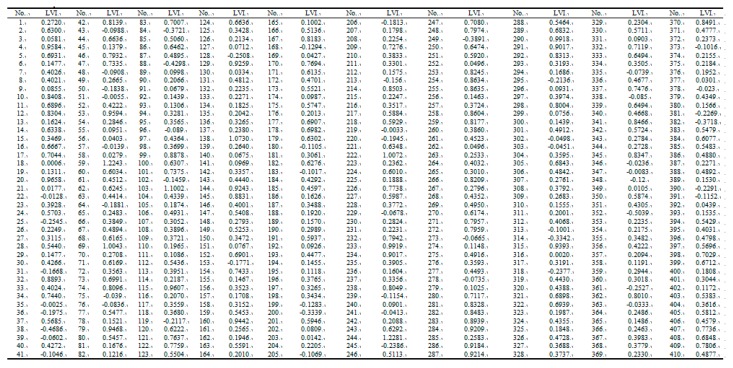
All livelihood vulnerability indices of the sample.

Livelihood vulnerability index is a relative concept, which reflects the trend of livelihood’s unstable development when the group faces policy shock or environmental change [40]. When vulnerability is positive, it shows that the higher the risk, the greater the exposure and sensitivity. Meanwhile, the larger the positive value is, the more vulnerable it is; when the vulnerability is negative, it means that its risk is lower, that is, its adaptability is stronger. The smaller the negative value is, the less vulnerable it is [41]. Combined with the survey data, it can be seen from the calculation of Formula (6) that different types of livelihood vulnerability have great differences. On the whole, the vulnerability index of the relocated employees is concentrated between −0.5 and 1.23 (Figure A2 in the Appendix A), which indicates that the individual risk index of the surveyed employees is higher, and their livelihoods are fragile.

**Figure 3:**
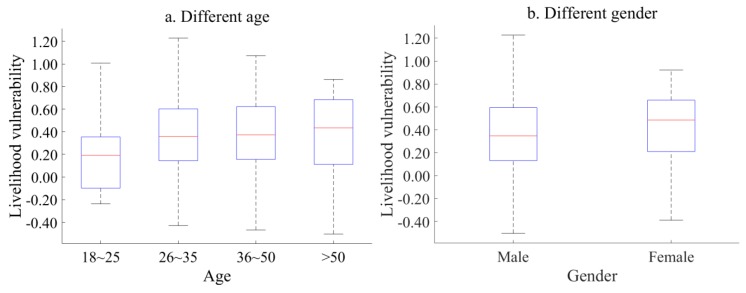
Livelihood vulnerability index for employees of different ages and genders.

There are also differences in livelihood vulnerability index among employees of different ages (Figure 3a). The 18–25 year old group’s livelihood vulnerability is low. The vulnerability index distribution of employees aged 26–35 is the most discrete. At the same time, the livelihood vulnerability of the 36–50 year old group is relatively high and concentrated.

According to Figure 3b, male and female employees show different degrees of livelihood vulnerability. Male’s vulnerability is more dispersed, which means that male employees have a trend of two-level differentiation when they are impacted by external forces. Moreover, the median of vulnerability index tends towards the lower quartile, indicating that the livelihood vulnerability index is low. However, women’s livelihood vulnerability is relatively concentrated, and the median of vulnerability index tends towards the upper quartile, indicating that the livelihood vulnerability index is generally high.

**Figure 4:**
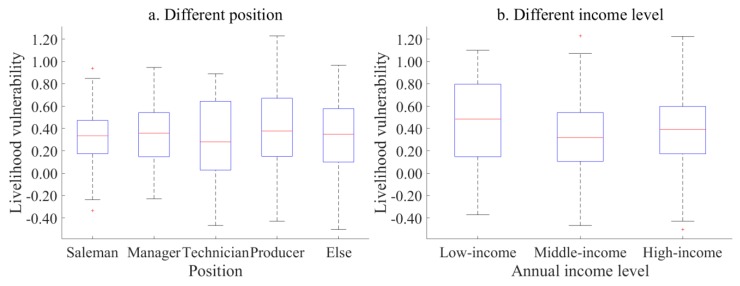
Livelihood vulnerability index for employees of different positions and income levels.

In Figure 4a, compared with the other four types of positions, the livelihood vulnerability index of producer is unevenly distributed and internally differentiated, which is related to their work attributes. However, the livelihood diversity of producers is low, and their wage accounts for a higher proportion of their total family income, which directly leads to a sharp decline in their income. The livelihood vulnerability of technicians, managers and salesmen is low.

The livelihood vulnerability index of employees from different income groups is shown in Figure 4b. It can be found that the median of the livelihood vulnerability index among high-income employees tends to the lower quartile, and there are specific values at both ends, indicating that the livelihood vulnerability of high-income employees is biased and relatively low-balanced. The median of the vulnerability index among middle-income employees is in the middle of the box, and the upper and lower cutoff points are close to the upper and lower quartiles, showing a standard distribution, indicating that the distribution of livelihood vulnerability of middle-income employees is relatively uniform. The livelihood vulnerability of low-income employees has a relatively high equilibrium. At the same time, the interquartile distance of middle-income employees is relatively narrow, and the specific value is greater, which indicates that the livelihood vulnerability index of this kind of employee is dispersed to two poles.

**Figure 5:**
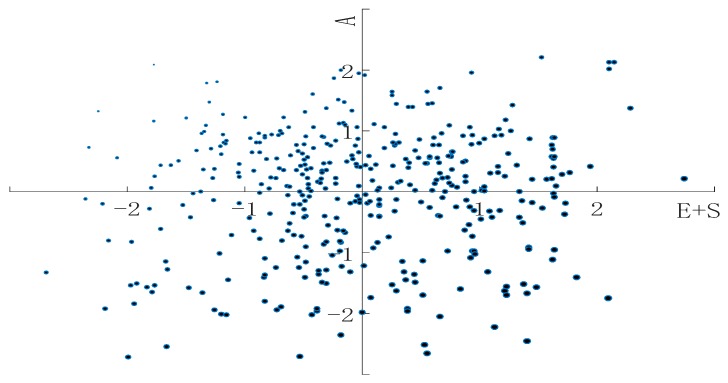
Classification of employee livelihood vulnerability in relocation enterprises.

Based on the Livelihood Vulnerability Assessment Model in Section 3.1.2, this study calculated the exposure index (*E*), sensitivity index (*S*), adaptability index (*A*) and livelihood vulnerability index (*LVI*) for each family in the sample, which were then used to classify families according to degree of livelihood vulnerability (Figure 4). The average values of the four indices were 0.5277, 0.3452, 0.5104 and 0.3625, respectively. To examine differences in risk, adaptability and livelihood vulnerability for the sampled families, we standardized the *E + S* and *A* to avoid the influence of magnitude. The horizontal and vertical axes in Figure 5 represent the standardized values of *E + S* and *A* respectively. Moreover, each single dot represents an employee’s family, and dot size represents the degree of livelihood vulnerability.

According to the distribution of dots in each quadrant, all sampled employees are classified into four types (Table 4): Type I represents high risk and high adaptability. The average values of this type are 1.1575 (*E + S*) and 0.5805 (*A*), which are higher than the other three types. The average value of *LVI* is 0.5770, which is moderate compared with other types. Type II represents low risk and high adaptability, with an average of 0.6389 (*E + S*) and 0.5794 (*A*). The risk index (*E + S*) is low, and the adaptability (*A*) is higher than other types, so the average *LVI* is 0.0595, which is the lowest. Type III represents low risk and low adaptability. The average value is 0.5925 (*E + S*) and 0.4088 (*A*). The risk index and adaptability are lower than in other types. In addition, the average value of *LVI* is 0.1837, which is in the middle compared with other types. Type IV represents high risk and low adaptability, with an average of 1.1383 (*E + S*) and 0.4138 (*A*). The risk index is high, while adaptability is relatively low, which leads to the highest *LVI* (0.7245) of the four types.

### 4.3. Influencing Factors of Employees’ Livelihood Vulnerability

Family characteristics, individual characteristics and other indicators are taken as explanatory variables, and the exposure, sensitivity, adaptability and livelihood vulnerability of employees to enterprise relocation are taken as explained variables. Multiple linear regression model is used to empirically analyze the influencing factors of employees’ livelihood vulnerability in relocation enterprises. The results are shown in Table 5.

The results demonstrate that the education of employees has a positive and negative correlation with adaptability and livelihood vulnerability at the statistical levels of 1% and 5%, respectively. It shows that, with the improvement in highest education, employees’ adaptability in relocation enterprises tends to increase, and their livelihood vulnerability tends to decrease. Family burden ratio has a significant negative impact on the statistical level of 5%, indicating that human capital stock has an important impact on reducing employees’ livelihood vulnerability.

In addition, annual income per capita, social network, housing convenience and understanding of policies all have significant negative effects on livelihood vulnerability. That is to say, when facing external changes such as risk impact, if employees have a high income, convenient living environment and transportation, and are connected more with emerging things and social public organizations, as well as understanding the relevant policies of our country, they will show stronger adaptability and lower livelihood vulnerability.

### 4.4. Influencing Factors of Employees’ Sustainable Livelihood Ability

To a great extent, the sustainable development of employees after relocation depends on the improvement of their livelihood strategies and the improvement of their overall quality and skills [21]. In other words, the sustainable livelihood ability is directly affected by the adaptability of the relocated employees to life after relocation and speed of adaptability’s transformation. In Table 4, types III and IV account for 41.22% of the total samples, which means nearly half of the relocated families have low adaptability. To determine the factors that affect the sustainable livelihood ability of employees, and measure the degree of obstacle specific factors pose to sustainable livelihood ability, this paper combines Equations (7) and (8) to calculate these factors, including: annual income per person, education, the burden ratio of the elderly, the burden ratio of children and housing area, etc.

According to Table 6, the first five of the above factors are selected as the obstacles to the sustainable livelihood ability of the relocated employees. In addition to range, the obstacles for type III employees are almost the same as for all employees with low sustainability. However, a new factor in type IV employees is the annual income per person. In general, the main obstacles to the sustainable livelihood of employees are living convenience, relocation adaptability, policy understanding, children’s burden ratio, education, and the annual income per person. Two key obstacles to maintain or improve sustainable livelihood ability are whether their current living environment is comfortable and convenience and whether they are used to it.

### 4.5. Case Study on Compensation Model of Relocated Employees

Taking two chemical relocation enterprises in the Hubei Province of the Yangtze River Basin as an example, 80 employees were sampled, and their relocation losses were measured using the compensation model constructed in Section 3.3. According to Section 4.3 and Section 4.4, the main factors that affect the livelihood vulnerability and sustainable livelihood ability of employees are: education, housing area, social network, annual income per person, children’s burden ratio and housing convenience. After classification, the variables of each factor are shown in Table 7.

#### 4.5.1. Determination of the Fixed Social Capital Compensation Standard

(1) The cost saving from education (*OC*_11_): The functional value of relocated employees’ education can be replaced by the cost when the employees enter the talent market to apply for jobs again. According to the survey, every household will search for work every year for three days and enter the talent market three times [42]. The daily missed work fee is 100 CNY, the transportation fee is 30 CNY, and the admission fee of the talent market is 10 CNY each time, so the cost of job-hunting is 420 CNY/year. The sample shows that 49.52% of the interviewees are looking for jobs through the talent market, so the cost saving from education is 207.98 CNY/year;
(2) The cost saving of social network (*OC*_12_): This includes the cost of technical training and the balance of human relationship income and expenditure. The cost of technical training can be replaced by training fees. According to the investigation, the minimum fee for skill training for urban employees is more than 600 CNY [43], so the annual fee for training here is 600 CNY. It is known that 24.88% of the surveyed employees obtain technology through social networks, so the cost of technical training saved by each household through social networks is 149.28 CNY every year. In order to maintain their own social network, employees of enterprises will spend a lot of money on some important events, such as marriage, childbirth, promotion of colleagues and passing exams. It will take a long time to balance the payments. Due to the relocation of enterprises and the separation of social networks and spaces, the long-term and balanced geographical relationship between the original closed and stable circle has been destroyed, meaning the important expenditure of enterprise employees cannot be paid back. In the past five years, the average annual personal expenses of the interviewed employees exceeded personal income by 1034 CNY.

Therefore, the annual fixed social capital compensation standard for each relocated employee in this region is

(14) $$\;OC_{1}=\overset{2}{\underset{i\;=\;1}{\sum }}OC_{1i}=207.98+149.28+1034=1391.25\;CNY\;$$

#### 4.5.2. Determination of the Variable Opportunity Cost Compensation Standard

In order to obtain the compensation standard of variable opportunity cost, we take the cost index level of employees as the dependent variable and the relevant influencing factors as the independent variable; at the same time, we fit the questionnaire data with multiple functions to get the measurement model, reflecting the relationship between the dependent variable and the independent variable, then the coefficient (*A_jk_*) of the influencing factor (*X_k_*) on each cost index level (*OC*_2*j*_, (*j* = 1, 2, 3)) can be estimated: *A*_11_ = 1.843, *A*_12_ = 0.651, *A*_23_ = 0.505, *A*_31_ = 0.002, *A*_34_ = 102.535. Through factor analysis, the measurement models of *OC*_24_ and its influencing factors are as follows

*F*_1_ = 0.492*x*_5_ + 0.272*x*_6_ + 0.552*x*_7_ − *x*_8_ + 0.325*x*_9_ (15)

*F*_2_ = −0.352*x*_5_ + 0.555*x*_6_ + 0.048*x*_7_ + 0.634*x*_8_ + 0.086*x*_9_ (16)

*OC*_24_ = 0.332 *F*_1_ + 0.219 *F*_2_ + 1.116 (17)

where *F*_1_ and *F*_2_ are the principal component of the influencing factor (*X_k_*).

In the same way, factor analysis can be conducted again according to the corresponding values of each variable after enterprise relocation, and the measurement model of *OC’*_24_ and its influencing factors can be obtained as follows

*F*_1_*’* = 0.251*x*_5_ + 0.148*x*_6_ + 0.285*x*_7_−*x*_8_ + 0.157*x*_9_ (18)

*F*_2_*’* = −0.183*x*_5_ + 0.288*x*_6_ + 0.023*x*_7_ + 0.332*x*_8_ + 0.045*x*_9_ (19)

*OC’*_24_ = 0.167 *F*_1_*’* + 0.183 *F*_2_*’* + 1.003 (20)

Based on this, it can be estimated that

(21) $$\;OC_{2}=\overset{4}{\underset{j\;=\;1}{\sum }}\left(OC_{2j}-OC_{2j}^{\prime }\right)=3735.09\;CNY\;$$

Then, *OC* = *OC*_1_ + *OC*_2_ = 5126.34 CNY/year, that is, *CS* ≥ 5126.34 CNY/year.

## 5. Discussion

### 5.1. Employees’ Livelihood Vulnerability

According to Section 4.2, the 18–25 year old group is young people just entering the workplace, with an age advantage. They not only have active thinking skills and a strong desire for new knowledge, but also have a strong ability to accept new things and master new skills, and can bear corresponding work pressure. Therefore, they have strong adaptability to the new environment after the relocation of enterprises. As a result, their livelihood vulnerability is low. The vulnerability index distribution of employees aged 26–35 is the most discrete. With the increase in age, some people will be less vulnerable because of their rich work experience and gradual proficiency of skills. However, according to the individual interviews, the “40, 50” personnel in the relocation enterprises are the most difficult group in the process of reemployment. As they grow older, their ability to acquire new knowledge and skills weakens. Once these employees lose their jobs, they will be in a more disadvantageous position in the re-employment or entrepreneurship market.

Combined with Figure 3b, Male employees are generally more optimistic than female employees when facing the relocation of enterprises. There is also evidence that incidence of abnormal psychology in females is higher than in males [44]. Females are more limited in finding a job and have a greater demand for job stability. They have a series of special periods (such as pregnancy, childbirth and menopause) and need to invest a lot of time in the family (such as caring for the elderly and children). This makes it more difficult for women workers to re-enter the workforce than men. In the process of enterprise transformation and upgrading, female employees are more likely to feel resistant to new things and changes in industry situation than male employees.

As for different positions, although producers can gain some compensation in the process of enterprise relocation, they will face a greater livelihood risk after relocation due to their low cultural quality and limited cognition of their skills. Their low livelihood diversity is because of the difficulty of reemployment, the change in interpersonal relationships, working environment and higher requirements for their working skills and environmental adaptability, which means producers have intense anxiety about enterprise relocation, and show higher livelihood vulnerability. However, technicians and managers are the technical backbone and core of enterprise; although the impact of policies such as enterprise relocation will have a certain impact on their livelihood, their rich working experience, skilled technical and professional knowledge of technicians, high organization and management ability will allow them to find relatively stable and high-income jobs soon after enterprise relocation. Difficulties in the reemployment of salesmen are relatively small because of their good language expression and communication ability and rich reserves of practical experience.

Through Figure 5 and Table 4, four types of livelihood vulnerability for all sampled employees are classified, which can indicate the differences in risk, adaptability and livelihood vulnerability for employees. Among the four types, 19.02% of the employees have high LVI, which shows that this type of employee is at a high risk when facing relocation. The reason for this is that they have a strong dependence on the work before relocation, their wages account for a large proportion of their family income and, as their living expenses are highly dependent on gross income, their livelihood ability cannot withstand external impact such as sudden unemployment, which directly leads to a large property loss after relocation, and most of them are more likely to borrow money. Meantime, the proportion of employees with a mid-range LVI is 50.98%, and the proportion of employees with a low LVI is 30.00%. This means that more than half of the relocated employees are in the middle range of the livelihood vulnerability index; they are greatly affected by the relocation, but have not received enough compensation and are unable to maintain their livelihood. Therefore, it is very important to make accurate compensation for them, focusing on their livelihood loss.

### 5.2. Influencing Factors of Employees’ Livelihood Vulnerability

From the Section 4.3, annual income per person, education, housing area, residential convenience and understanding of enterprise relocation policy can significantly influence employees’ livelihood vulnerability. Employees’ cognitive level and skills represent their learning ability, their education, and their knowledge acquisition and transformation abilities, which are all conducive to enhancing employees’ ability to cope with external shocks. This often makes it easier to obtain jobs and adjust their own state, eliminate the vulnerability impact brought by the external environment, and maintain the good operation of their livelihood system.

In the meantime, convenient living conditions can bring employees good life experience and life satisfaction, and improve the life quality of employees. Their risk of being affected by relocation will be naturally reduced, and their livelihood vulnerability will be greatly reduced. Employees who pay attention to ecological and environmental protection policies can understand the trends in national environmental protection policies better, and better predict and understand the measures taken by the state for environmental protection, so as to grasp changes in policy and stay ahead of the policies.

In addition, family burden radio has a significant negative impact on livelihood vulnerability, indicating that human capital stock has an important impact on reducing employees’ livelihood vulnerability, which may be related to the family structure in China. In China’s main families (often with three generations including grandparents and grandchildren), the financial reserves of the elderly can be used for all family members to share, so the high burden ratio of the elderly can significantly reduce the livelihood vulnerability of employees, while improving the adaptability of the whole employee family. However, the results show that trust in people increases the livelihood vulnerability of employees. The more trust employees have in the people around them, the more dependent they are on the help of others. When faced with the impact of external forces such as relocation, the emotional ties with the surrounding people will be greatly affected by the fracture, which directly leads to an increase in their livelihood vulnerability.

### 5.3. Compensation Model of Relocated Employees

In the past research of compensation mechanism, the compensation object is the famers, or the residents who have lost their land or houses. However, due to the relocation of chemical enterprises, the employees temporarily or permanently lost job opportunities that could help them achieve social and self-worth, as well as their original social network, and have to face a succession of livelihood challenges, such as the transfer of living space, the loss of economic sources and society capital, and an increase in living costs. In order to make up for the loss effectively and improve the sustainable livelihood ability of employees after relocation, it is imperative to explore a kind of accurate compensation standard. However, there are few quantitative studies on the compensation of employees in environmental relocation enterprises, which makes the determination of compensation standard lack a quantitative basis. This means that relocated employees cannot get economic compensation.

In order to deal with this from the perspective of urban employees suffering from non-natural losses, based on the measurement of livelihood vulnerability, alternative opportunity cost method is used to determine the compensation standard in this paper. According to Section 4.5, the minimum compensation level for this special group is 5126.34 CNY/year. This provides a new idea for the compensation mechanism of similar situations in developing countries and even the world. In addition, although access to employment information will become more and more market-oriented with the deepening of the market, studies in various countries show that, due to the asymmetry of information, even in developed countries, social networks are still an important channel for access to information [45].

### 5.4. Limitations

This paper has the following limitations: (1) in this paper, the relocation of employees as a whole is studied, but there is no further classification of the group and differential compensation and resettlement. (2) The compensation standard designed in this paper calculates the lowest level of compensation. The specific compensation mechanism, as well as the resettlement mode for different employees, have not been included in this paper.

According to the above limitations, in future research, the targeting of such special groups, and the further designing of different resettlement strategies according to different types of targeted employees can be a focus. In addition, in view of special groups such as employees in relocated enterprises, this paper tries to design a special method for its compensation lower limit. Then, how to further perfect this compensation mode and form a complete compensation mechanism will be the direction of future research.

## 6. Conclusions and Suggestions

### 6.1. Conclusions

Based on the survey data of 410 employees in the relocation enterprises in Hubei Province of the Yangtze River Basin, this study pointed out the differences between the special group and groups affected by environmental protection in the past, analyzed the external impact and livelihood vulnerability characteristics of the employees using “Exposure-Sensitivity-Adaptability” analysis framework of IPCC, and found out the core factors that affect the livelihood vulnerability and sustainable livelihood ability of the employees. Based on these, a new compensation standard calculation method for this special group is explored to alleviate their livelihood vulnerability. The conclusions show that:

(1) On the whole, with the increase in age, the livelihood vulnerability index presents a gentle, inverted U-shaped trend. Employees of all ages show a certain degree of livelihood vulnerability;
(2) There are differences in livelihood vulnerability between male and female employees—women’s livelihood vulnerability is relatively concentrated and generally high;
(3) The livelihood vulnerability of producers is relatively high, and the vulnerability index is unevenly distributed and internally differentiated;
(4) The key obstacle factors affecting the sustainable livelihood of families are: living convenience, adaptability to relocation, policy understanding, children’s burden ratio, education, and annual income per person. If the current living environment is more comfortable and convenient, they will show a lower livelihood vulnerability and higher sustainable livelihood capacity;
(5) In view of the livelihood vulnerability of urban employees in environmental protection relocation enterprises, this paper designs a new compensation standard calculation model— Alternative Opportunity Cost Method—which can better reflect the compensation effect of the opportunity cost within the definition of international existing compensation mechanisms and realize the leap from concept to action.

### 6.2. Suggestions

Based on the above conclusions, this paper makes the following suggestions: (1) the government and enterprises could improve the knowledge and skills of their employees through various means, such as expanding education investment, carrying out differentiated training for different types of employees, providing entrepreneurial employment and so on. (2) Create an information-sharing platform to improve the adaptability of employees to the new environment. On the basis of maintaining the original social network, promote the social integration of employees in the new environment, and further improve and build the social relationship network of relocated employees, effectively improve the social relationship of employees and accumulate the human capital of employees. (3) Through the integration of relocation resources, separate development support projects could be set up in the financial transfer payment allocation at all levels to fully tap the endogenous power of the employees in the relocation enterprise, and actively guide the employees to achieve self-development. (4) Establish corresponding laws and regulations, standardize social welfare systems such as pension and medical care after retirement for employees, and guarantee employment for employees in cross-regional and industry positions. (5) For special groups such as employees in relocated enterprises, the government needs to take the lost social capital and development opportunity costs as the basis for the calculation of compensation standards, and formulate differentiated standards to allocate compensation funds, which is conducive to improving the efficiency of compensation in social capital.

## Figures and Tables

**Table 1:** Evaluation index system of livelihood vulnerability.

Dimensions	Indices	Weights ^1^	Meaning and Assignment of Indices	Mean	Standard Deviation
Expose (E)	Property loss (E_1_)	0.0250	Amount of personal property damage caused by enterprise relocation/CNY	28,825.27	20,469.92
	Credit possibilities (E_2_)	0.0300	Possibility of staff requiring credit after relocation. Sure = 1, larger = 2, generally = 3, smaller = 4, no = 5.	2.83	1.48
	Housing situation (E_3_)	0.0524	Rent = 1, rural self-house = 2, urban commercial house = 3	2.36	0.71
Sensitivity (S)	Health (S_1_)	0.0159	Medical expenses accounted for less than 20% of total household income = 1, 20%–50% = 2, more than 50% of total household income = 3.	1.53	0.66
	Negative effects of relocation (S_2_)	0.0475	Number of options for investigating the negative impact of relocation on employees	1.93	0.98
	Income dependence (S_3_)	0.0066	The proportion of enterprise wage income to family income	0.70	0.26
	Dependence on living expenses (S_4_)	0.0035	The proportion of household general living expenditure to total household expenditure	0.50	0.23
Adaptability (A)	Annual income per person (A_1_)	0.0608	Annual income per person in family/CNY	34,232.68	20,940.80
	Education (A_2_)	0.0797	Junior high school and below = 1, secondary or high school = 2, tertiary or undergraduate = 3, graduate above = 4	2.45	0.61
	Old age burden ratio (A_3_)	0.0407	Number of elderly people over 60 years of age	1.69	1.23
	Children’s burden ratio (A_4_)	0.0627	Number of children under 15	0.75	0.63
	Housing area (A_5_)	0.0608	Household housing area/m^2^	108.62	24.89
	Credit capital (A_6_)	0.0098	In the past three years, whether there has been any experience of borrowing money (banks, small loan companies, relatives and friends, etc.); yes = 1, no = 0.	0.64	0.48
	Skills training (A_7_)	0.0326	Yes = 1, no = 0	0.84	0.37
	Trust in people around (A_8_)	0.0996	Very distrust = 1, comparative distrust = 2, generally = 3, comparative trust = 4, very trust = 5	3.62	0.77
	Social network (A_9_)	0.0215	Number of civil servants among relatives	0.97	1.87
	Self-assessment of adaptability for relocation (A_10_)	0.1173	Incapacity = 1, low ability = 2, medium ability = 3, relatively high ability = 4, high ability = 5	3.13	0.77
	Residential convenience (A_11_)	0.1111	Yes = 1, no = 0	0.68	0.47
	Understanding of Enterprise Relocation Policy (A_12_)	0.1157	Very not understanding = 1, comparative not understanding = 2, generally = 3, comparative understanding = 4, very understanding = 5	3.49	0.89
	Livelihood Diversity (A_13_)	0.0068	Number of livelihood activities of employee families	1.43	0.63

^1^ Weight calculation is based on principal component analysis (PCA), which is mentioned in Section 3.1.2.

**Table 2:** Descriptive statistics of relocated employees of chemical enterprises in the Yangtze River Basin.

Statistical Items		Number	Percentage %	Statistical Items		Number	Percentage %
Gender	Male	346	84.39	Education	Junior high school and below	21	5.12
	Female	64	15.61		Secondary or high school	186	45.37
Age	18~25	19	4.63		Diploma or undergraduate	199	48.54
	26~35	188	45.85		Postgraduate and above	4	0.98
	36~50	182	44.39	Number of families	1~3	117	28.54
	Over 50	21	5.12		4~6	261	63.66
Position	Salesman	40	9.76		7 and above	32	7.80
	Manager	54	13.17	Annual income per person (CNY)	10,000 and below	83	20.24
	technician	48	11.71		10,000~50,000	183	44.63
	Producer	201	49.02		50,000 and above	144	35.12
	Else	67	16.34				

**Table 3:** Descriptive statistics of relocated employees of chemical enterprises in the Yangtze River Basin (by position classification).

Position	Number of Families	Number of Families Over 60 Years Old	Number of Families under 15 Years Old	Annual Income Per Person (CNY)	Housing Area (m^2^)
Salesman	3.75	2.08	0.63	46,800.00	103.33
Manager	4.26	1.57	0.78	37,583.33	114.26
technician	4.65	1.56	0.73	40,104.17	109.60
Producer	4.72	1.70	0.81	28,653.73	108.72
Else	4.42	1.58	0.67	36,559.70	106.20

**Table 4:** Types of livelihood vulnerability of employees in sample relocation enterprises.

Type	(E + S)		(A)		(LVI)		Sample	
	Attribute	Mean	Attribute	Mean	Attribute	Mean	Number	Percent
I	High	1.1575	High	0.5805	Middle	0.5770	118	28.78%
II	Low	0.6389	High	0.5794	Low	0.0595	123	30.00%
III	Low	0.5925	Low	0.4088	Middle	0.1837	91	22.20%
IV	High	1.1383	Low	0.4138	High	0.7245	78	19.02%
Total	-	0.8729	-	0.5104	-	0.3625	410	100%

**Table 5:** Estimated results of influencing factors on livelihood vulnerability of employees in relocated enterprises.

Variables	E	S	A	LVI
Gender	0.012541 (0.014876)	−0.059302 * (0.034018)	−0.002832 (0.002413)	−0.043929 (0.037558)
Age	0.008224 (0.008147)	−0.021641 (0.018631)	−0.000709 (0.001321)	−0.012708 (0.020569)
Annual income per person	2.28 × 10^−8^ (2.49 × 10^−7^)	−7.26 × 10^−7^ (5.69 × 10^−7^)	7.61 × 10^−7^ *** (4.03 × 10^−8^)	−1.46 × 10^−6^ ** (6.28 × 10^−7^)
Education	−0.007789 (0.009368)	0.010760 (0.021464)	0.031304 *** (0.001520)	−0.049853 ** (0.023653)
Old age burden ratio	−0.004780 (0.004434)	0.002863 (0.01014)	0.008381 *** (0.000719)	−0.016024 ** (0.011195)
Children’s burden ratio	−0.009361 (0.008856)	−0.020553 (0.020252)	0.016794 *** (0.001436)	−0.046707 ** (0.022359)
Housing area	0.000284 (0.000212)	−0.001237 ** (0.000484)	0.000424 *** (0.000034)	−0.001377 *** (0.000535)
Trust in people around	0.017488 ** (0.007317)	0.024142 (0.016733)	0.033005 *** (0.001187)	0.008625 ** (0.018474)
Social network	−0.003407 (0.002667)	−0.013168 ** (0.006098)	0.001940 *** (0.000433)	−0.018414 *** (0.006733)
Self-assessment of relocation adaptability	0.005287 (0.006921)	−0.000868 (0.015828)	0.033270 *** (0.001123)	−0.028851 * (0.017474)
Residential convenience	0.036357 *** (0.011383)	−0.033057 (0.026032)	0.140885 *** (0.001846)	−0.137586 *** (0.028742)
Understanding of enterprise relocation policy	0.011409 * (0.006333)	−0.022437 (0.014482)	0.035590 *** (0.001027)	−0.046618 *** (0.015989)
Livelihood diversity	0.035010 *** (0.008140)	−0.036123* (0.018615)	0.004872 *** (0.001320)	−0.005984 (0.020551)
Number of families	−0.008838 ** (0.004201)	0.022550 ** (0.009608)	0.000630 (0.000681)	0.013082 (0.010607)
Position	0.015718 *** (0.004629)	0.012566 (0.010586)	0.000712 (0.000751)	0.027572 ** (0.011687)
R^2^	0.8195	0.1066	0.9777	0.4586
F	111.48	2.93	1076.87	20.80

Note: Standard deviation is in parentheses; ***, ** and * are significant at 1%, 5% and 10% statistical levels, respectively, calculated by software Stata14.

**Table 6:** The factors and degree of obstacles to employees’ sustainable livelihood ability in relocation enterprises.

Rank	Employees with Low Adaptability		Category III Employees		Category IV Employees	
	Obstacle Factors	Obstacle Degree	Obstacle Factors	Obstacle Degree	Obstacle Factors	Obstacle Degree
1	Residential convenience	0.1581	Residential convenience	0.1564	Residential convenience	0.1602
2	Relocation adaptability	0.1327	Relocation adaptability	0.1357	Relocation adaptability	0.1291
3	Understanding of enterprise relocation policy	0.1160	Understanding of enterprise relocation policy	0.1148	Understanding of enterprise relocation policy	0.1173
4	Children’s burden ratio	0.1129	Children’s burden ratio	0.1104	Children’s burden ratio	0.1158
5	Education	0.0913	Education	0.0923	Annual income per person	0.0908

**Table 7:** Variable descriptions.

Variables	Variable Meanings	Variable Descriptions
OC_11_	Cost savings from education	The cost for employees to enter the talent market and apply for jobs again
OC_12_	Cost saving of social network	Including the cost of technical training and the balance of human relationship income and expenditure
OC_21_	Housing area	The main influencing factor is the annual income of the family
OC_22_	Annual income per person	The main influencing factor is salary
OC_23_	Children’s burden ratio	The cost can be replaced by the education investment of the staff to the children. The main influencing factors include the annual income of the family and the education of the parents
OC_24_	Living convenience	The main influencing factors include shopping convenience (number of supermarkets and shopping malls nearby), medical convenience (distance to hospital), transportation convenience (distance to bus station, downtown and workplace)
